# Identification of a Specific Role of Dihydrozeatin in the Regulation of the Cell Differentiation Activity in *Arabidopsis* Roots

**DOI:** 10.3390/plants14101501

**Published:** 2025-05-16

**Authors:** Federico Vinciarelli, Mirko De Vivo, Alessio Terenzi, Francesca Cazzaniga, Samuele Amati, Pierpaolo Damato, Elena Salvi, Marta Del Bianco, Riccardo Di Mambro, Paolo Costantino, Sabrina Sabatini, Raffaele Dello Ioio

**Affiliations:** 1 Department of Biology and Biotechnology “Charles Darwin”, Sapienza University of Rome, 00185 Rome, Italy; federico.vinciarelli@uniroma1.it (F.V.); mirko.devivo@uniroma1.it (M.D.V.); francesca.cazzaniga@uniroma1.it (F.C.); samuele.amati@uniroma1.it (S.A.); damato.2000949@studenti.uniroma1.it (P.D.); paolo.costantino@uniroma1.it (P.C.); 2 Department of Biology, Università di Pisa (UNIPI), 56126 Pisa, Italy; alessio.terenzi@phd.unipi.it (A.T.); elena.salvi@unipi.it (E.S.); riccardo.dimambro@unipi.it (R.D.M.); 3 Agenzia Spaziale Italiana (ASI), 00133 Rome, Italy; marta.delbianco@asi.it

**Keywords:** cytokinins, root development, root meristem, dihydrozeatin, hormonal regulation

## Abstract

The plant hormones cytokinins are a class of heterogeneous active compounds that control multiple aspects of development and physiology. Among cytokinins, trans-zeatin (*tZ*), the most abundant cytokinin, has been extensively studied in relation to its effects on development, and it plays a key role in promoting cell differentiation. In analogy with *tZ*, here we demonstrate that dihydrozeatin (DHZ) controls (root) development by promoting cell differentiation. By means of pharmacological and genetic analysis, we demonstrate that DHZ is specifically and uniquely perceived by the histidine kinase (HK) receptor AHK3, and that this interaction is sufficient to promote cell differentiation in the root meristem via activation of the transcription factors *ARABIDOPSIS* RESPONSE REGULATOR 1, 12, and 11. We also show that DHZ and *tZ* activity might be conserved among plants. Our results support the idea that different types of cytokinins act via specific receptors to exert their roles and suggest new approaches to study their activity in differentiation.

## 1. Introduction

Cytokinins (CKs) are key regulators of plant development. They can modulate the activity and maintenance of meristems in response to environmental cues (such as hydrotropism) and mediate the response to various stresses, such as osmotic stress [1,2,3,4,5,6,7,8,9,10]. CK are N6-prenylated adenine derivatives that differ in their isoprenoid or aromatic side chains [11,12]. Isoprenoid CKs are predominant in higher plants, trans-zeatin (*tZ*) and isopentenyladenine (iP) being the most abundant compounds of this subclass [13,14,15]. Other isoprenoid cytokinins have been isolated, but their activity and relevance in plants remain unexplored [16,17,18,19]. Among these, dihydrozeatin (DHZ) is a zeatin with a saturated side chain that derives from the reduction of *tZ* by zeatin reductase, and its activity in development has not been widely studied [20,21]. The perception and signal transduction of cytokinins consists of a multistep two-component system phosphotransfer cascade [2,22]. The involved receptors are transmembrane histidine kinases, and three of these are encoded in the *Arabidopsis thaliana* genome: *ARABIDOPSIS HISTIDINE KINASES 2–4 (AHK2*, *AHK3*, and *AHK4/CRE1)*, with AHK3 and AHK4/CRE1 being mostly involved in root patterning and development [23,24]. AHK3 and AHK4/CRE1 show partially overlapping expression patterns and redundant functions [23,25,26,27,28]. In vitro ligand binding assays demonstrated that the affinity of these two receptors for *tZ* is comparable, with AHK3 showing higher sensitivity to pH variations and higher affinity for DHZ than AHK4/CRE1 [29]. An in vivo confirmation of these results is still lacking, as is the demonstration of the activity of DHZ in (root) development, in contrast to the well-documented *tZ*.

In *Arabidopsis* root development, a set of stem cells located at the root tip generates transit amplifying cells that establish all the tissues that compose the meristem [30,31,32,33]. After a series of divisions, these cells cease to divide and start to elongate, entering the elongation zone of the root. The region between the meristematic and the elongation zones is the transition zone (TZ), a developmental boundary where cells stop dividing and start to differentiate [34,35,36,37]. It has been shown that *tZ* plays a crucial role in driving root meristem cells to differentiation, antagonizing auxin activity. At the Transition Zone (TZ), *tZ* is perceived by the AHK3 receptor, initiating a phosphorylation cascade that enters the nucleus and activating the transcription factors *ARABIDOPSIS* RESPONSE REGULATOR 1 and 12 (ARR1 and ARR12). In turn they induce the expression of *SHORT HYPOCOTIL 2 (SHY2)*, a member of the auxin/indole-3-acetic acid (Aux/IAA) gene family [38,39,40,41,42] involved in auxin signaling repression, and of the *GRETCHEN HAGEN 3.17 (GH3.17)* gene, which conjugates auxin with amino acids in the lateral root cap [30,34,43]. Transcriptional activation of these genes by *tZ* generates a minimum of auxin activity that prompts cells to exit from the meristem [34,44]. In addition to inhibition of auxin activity, it has been proposed that *tZ* induction of ARR1 promotes cell differentiation regulating the expression of *KIP-RELATED PROTEIN 2 (KRP2)*, a gene encoding a negative regulator of the cell cycle [44,45], and of α-*EXPANSIN 1 (EXPA1)* encoding for an enzyme involved in cell expansion [46,47,48,49,50,51,52]. Thus, *tZ* promotes the exit of cells from the meristem, allowing those cells to start the differentiation programs [53,54,55].

Via a genetic, pharmacological, and molecular analysis, we show that, besides *tZ*, DHZ also promotes cell differentiation, and we demonstrate that, in vivo, the AHK3 receptor is specifically dedicated to perceive DHZ. We also propose that DHZ, via the AHK3 receptor, activates the ARR1, 11, and 12 transcription factors to induce root cell differentiation. Finally, we suggest that the activity of DHZ is potentially conserved across plants.

## 2. Results

### 2.1. DHZ Promotes Cell Differentiation in Arabidopsis Root

**Figure 1:**
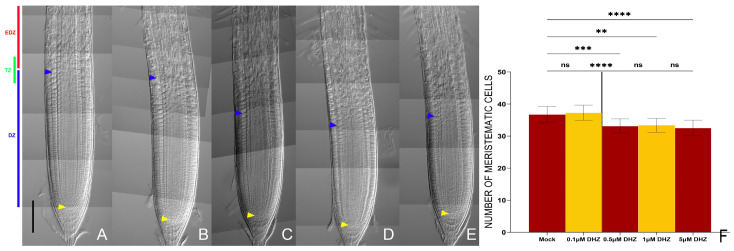
DHZ regulates root meristem size in a similar fashion to *tZ.* (**A**–**E**) Representative DIC optical microscope images of 6 dpg Wt plants. The roots were mock-treated with EtOH (**A**), or treated for 16 h with 0.1 (**B**), 0.5 (**C**), 1 (**D**), and 5 (**E**) μM DHZ. Yellow arrows indicate the QC, blue arrows indicate the last cortical cell of the meristem. Scalebar = 100 μm. In (**A**), a schematic representation of root zonation is depicted: in red, the elongation and differentiation zone (EDZ); in green, the transition zone (*TZ*); in blue, the division zone (DZ). (**F**) Analysis of meristematic cortical cell number of Wt (*Col-0*) plants treated for 16 h with 0.5 μM DHZ. Mock treatment: EtOH. Error bars indicate standard deviation (SD). (ns) indicates a *p*-value > 0.05, (**) indicates a significance with a *p*-value < 0.01, (***) indicates a significance with a *p*-value < 0.005, (****) indicates a significance with a *p*-value < 0.001, Kruskal–Wallis test with Dunn’s multiple comparisons post hoc test, N = 3, n = 20.

**Figure 2:**
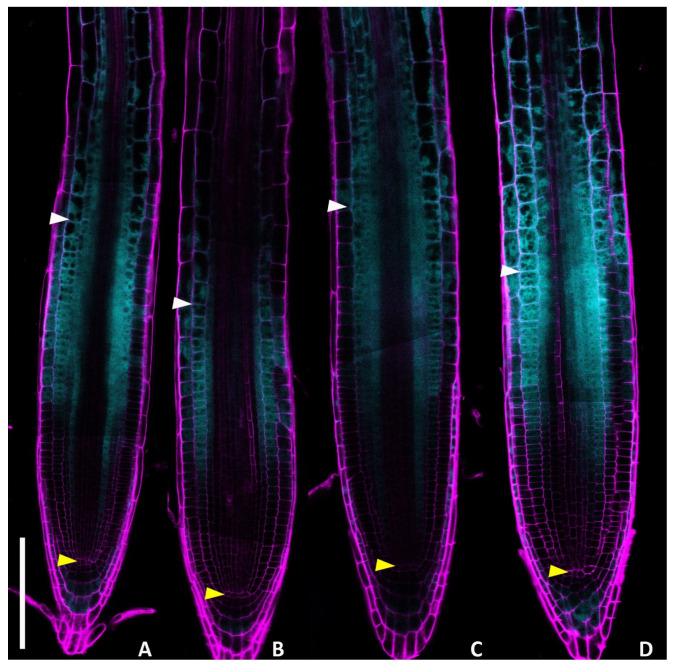
DHZ positions the TZ in a similar fashion to *tZ*. Confocal microscope images of 6 dpg roots expressing the *pRCH2::3xYFP* marker. Magenta and cyan pseudocolors represent propidium iodide (PI) staining and the YFP-related signal, respectively. In (**A**,**C**), the roots were mock-treated with DMSO and EtOH for 16 h; in (**B**,**D**), they were treated with 0.5 μM *tZ* and 0.5 μM DHZ for 16 h. Representative images were chosen for every condition. Yellow triangles indicate the QC; white ones, the last meristematic cortex cell. Note changes in *TZ* position. N = 3, n = 10. Scalebar = 100 μm.

To evaluate whether DHZ controls root development, we exposed 5-day-old *Arabidopsis* seedlings to various concentrations (0.1, 0.5, 1, and 5 μM) of exogenous DHZ. We observed that the 0.5 μM DHZ treatment was sufficient to reduce root length (Supplemental Figure S1). Hence, we compared the root meristem size (the average number of cortical cells within the meristem) of DHZ-treated plants with untreated controls (Figure 1). We noticed that the 0.5 μM treatment was sufficient to reduce the root meristem size, shifting the TZ position toward the stem cell niche, as also visualized by the root-ward shift of the localization of the TZ fluorescent reporter *RCH2::3xYFP* (Figure 1 and Figure 2). The effect of DHZ treatment appeared independent of hormone concentration within the tested range, as no significant change in meristem size was observed between the 0.5 and 5 μM DHZ treatments (Figure 1). Therefore, we decided to use a 0.5 μM concentration for the subsequent experiments.

**Figure 3:**
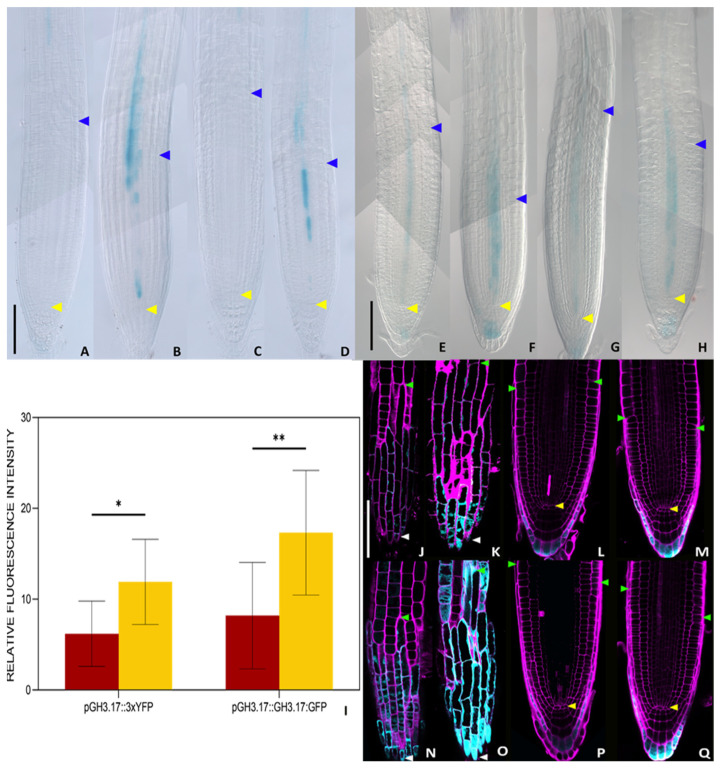
DHZ promotes cell differentiation, inducing pro-differentiation cytokinin targets. (**A**–**D**) Histochemical GUS assay of *Arabidopsis* roots. Representative DIC optical microscope images of 6 dpg transgenic plants carrying a *pSHY2::SHY2:GUS* fusion. N = 3, n = 15. (**E**–**H**) Histochemical GUS assay of *Arabidopsis* root. Representative DIC optical microscope images of 6 dpg transgenic plants carrying a *pKRP2::KRP2:GUS* fusion. N = 3, n = 15. Roots in (**A**) and (**E**) were mock treated with DMSO as a control for the *tZ* treatment, in (**B**) and (**F**) with 0.5 μM *tZ* for 16 h, (**C**) and (**G**) mock treated with EtOH as a control for the DHZ treatment, (**D**) and (**H**) with 0.5 μM DHZ for 16 h. Yellow arrows indicate the QC, blue arrows indicate the last cortical cell of the meristem. N = 2, n = 15. Scalebar = 100 μm. (**I**) Relative fluorescence quantification of the roots depicted in L-S. Only the cyan channel signal was quantified, selecting only cells of the lateral root cap (LRC), the only tissue in which GH3.17 is active as shown by [22,24]. Error bars indicate standard deviation (SD). N = 3, n = 10, Two-way ANOVA with Tukey’s post hoc test, (*) indicates a significance with a *p*-value < 0.05, (**) indicates a significance with a *p*-value < 0.01. (**J**–**M**) Confocal microscope images of 6 dpg roots expressing the *pGH3.17::3xYFP* construct. In (**J**) and (**K**), the focal plane was on the LRC, while in (**L**) and (**M**), it was on the meristem. The LRC plane was used for quantification (**I**). Magenta pseudocolor represents propidium iodide (PI) staining, while cyan represents one YFP-related signal. Only the YFP channel was used for quantification. In (**J**) and (**L**), the roots were mock-treated with EtOH for 16 h in (**K**) and (**M**), they were treated with 0.5 μM DHZ for 16 h. Representative images were chosen for every condition. White triangles indicate the root tip, yellow ones the QC, and green ones the uppermost LRC cell. Scalebar = 100 μm. (**N**–**Q**) Confocal images of 6 dpg roots expressing the *pGH3.17::GH3.17:GFP* construct. In (**N**) and (**O**), the focal plane was on the LRC, while in (**P**) and (**Q**), it was on the meristem. The LRC plane was used for quantification (**I**). Magenta pseudocolor represents Propidium Iodide (PI) staining, while cyan represents a GFP-related signal. Only the GFP channel was used for quantification. In (**N**) and (**P**), the roots were mock-treated with EtOH for 16 h in (**O**) and (**Q**) they were treated with 0.5 μM DHZ for 16 h. Representative images were chosen for every condition. White triangles indicate the root tip, yellow ones the QC, and green ones the uppermost LRC cell. Scalebar = 100 μm.

Cytokinin activity promotes cell differentiation in root tissues by activating *GH3.17* in the lateral root cap, and *KRP2* and *SHY2* in the vascular tissue [34,38,44]. To understand whether, as the *tZ*, also the DHZ induces the expression of these genes, we analysed the expression of the GUS transcriptional reporters of *SHY2* and *KRP2* (*pSHY2::GUS*, *pKRP2::GUS*) in DHZ-treated plants. In standard conditions, GUS activity was detectable in the vasculature from the TZ upward for both *pSHY2::GUS* and *pKRP2::GUS*. A 16-h DHZ treatment was sufficient to induce *SHY2* and *KRP2* expression in the vascular tissue of the meristem (Figure 3A–H). Furthermore, confocal microscopy analysis of *pGH3.17::3xYFP* lines and the translational fusion *pGH3.17::GH3.17:GFP* revealed an increased GH3.17 fluorescence signal after 16 h of DHZ treatment (Figure 3). Overall, these data suggest that DHZ controls meristem activity by acting in the canonical *tZ* pathway, passing by *KRP2*, *SHY2*, and *GH3.17*.

### 2.2. DHZ Is Perceived at the TZ by AHK3, but Not by CRE1/AHK4

**Figure 4:**
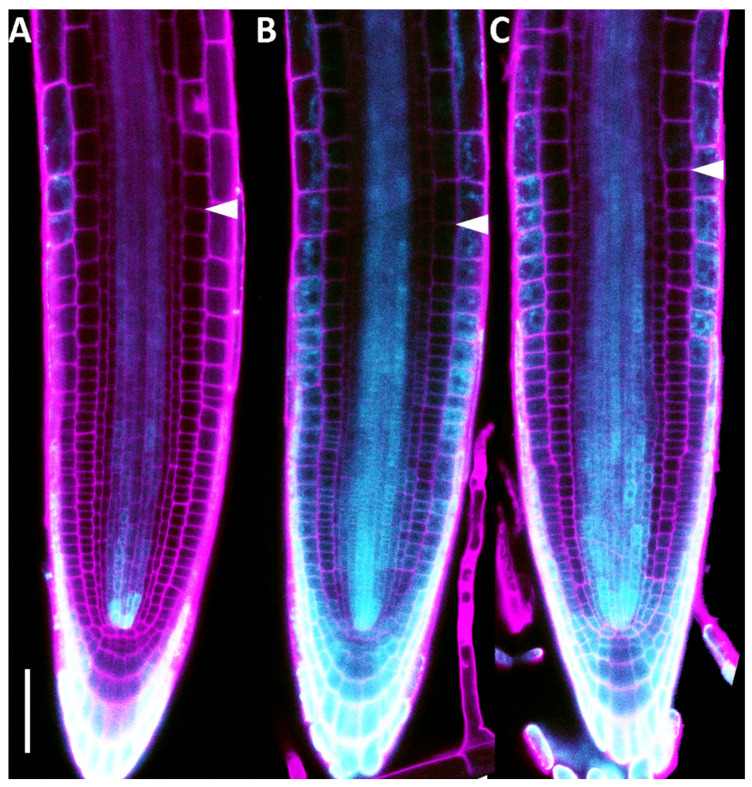
DHZ and *tZ* treatments enhance cytokinin activity in the root meristem. (**A**–**C**) Representative confocal microscope images of 5 dpg *TCS::nGFP* expressing plants, mock-treated (**A**) or treated with *TZ* 5 μM for 4 h (**B**) and DHZ 5 μM for 4 h (**C**). Magenta pseudocolor represents propidium iodide (PI) staining, while cyan represents a GFP-related signal. Scalebar = 100 μm. White arrowheads indicate the last meristematic cortical cell and hence the *TZ*. N = 3, n = 15.

To test whether DHZ is active in the TZ, we treated plants harbouring the cytokinin activity sensor *TWO COMPONENT SYSTEM* (*TCS::GFP*) with DHZ. In untreated roots, cytokinin activity is observed in the columella cells, lateral root cap, and vascular bundle (Figure 4A). After 5 h of DHZ treatment, the GFP signal is detectable in the epidermis and in the differentiated vascular bundle of the transition zone, resembling the response to *tZ* treatments (Figure 4B,C). These results suggest that DHZ is perceived at the transition zone similarly to *tZ*.

**Figure 5:**
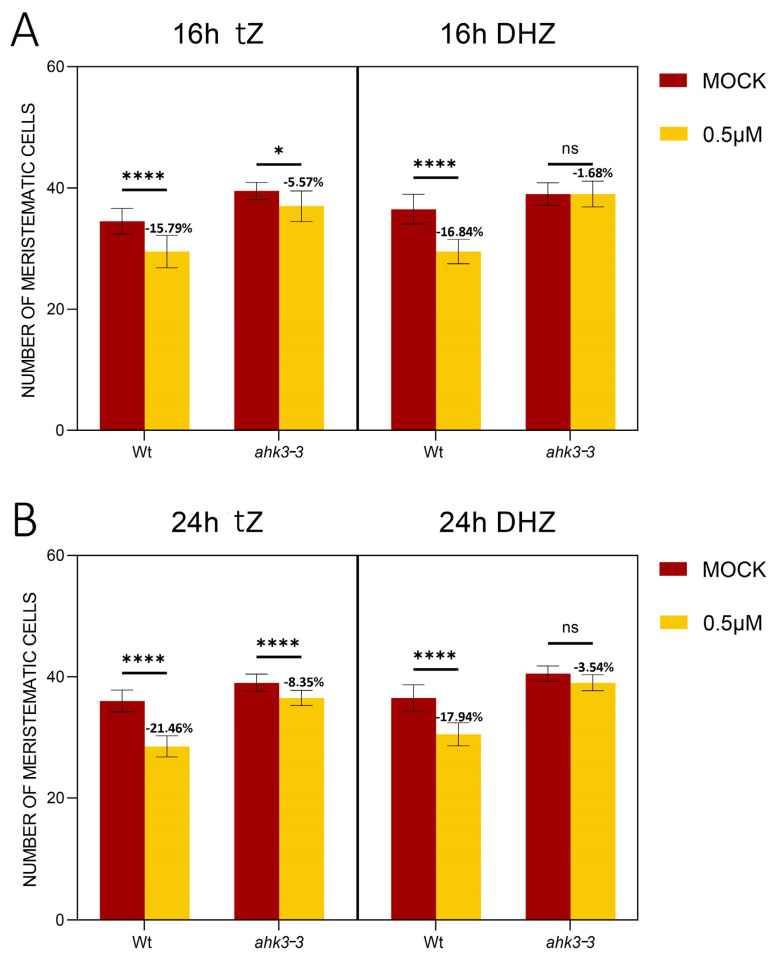
AHK3 mediates DHZ activity in the root meristem. (**A**) Analysis of meristematic cortical cell number of Wt and *ahk3-3* plants treated for 16 h with 0.5 μM *tZ* or 0.5 μM DHZ. Mock treatments: DMSO for *tZ*, EtOH for DHZ. Error bars indicate standard deviation (SD). (ns) indicates a *p*-value > 0.05, (*) indicates a significance with a *p*-value < 0.05, (****) indicates a significance with a *p*-value < 0.001, two-way ANOVA with Tukey’s post hoc test, N = 3, n = 15. Representative root images are reported in the Supplemental Figure S3. (**B**) Analysis of meristematic cortical cell number of Wt and *ahk3-3* plants treated for 24 h with 0.5 μM *tZ* or 0.5 μM DHZ. Mock treatments: DMSO for *tZ*, EtOH for DHZ. Error bars indicate standard deviation (SD). (ns) indicates a *p*-value > 0.05, (****) indicates a significance with a *p*-value < 0.001, two-way ANOVA with Tukey’s post-hoc test, N = 3, n = 15. Representative root images are reported in the Supplemental Figure S3.

Biochemical and heterologous expression experiments have shown that DHZ preferentially binds to the AHK3 receptor, while *tZ* is bound by both AHK3, CRE1, and AHK2 [29]. We hypothesized that DHZ perception by AHK3, similarly to *tZ*, activates a downstream molecular circuit controlling root meristem size. To test this, we exposed the roots of the *ahk3-3* loss-of-function mutant to DHZ. The *ahk3-3* mutants have larger meristems and longer roots compared to wild-type (Wt) plants (Figure 5 and Supplemental Figures S1–S3). Interestingly, the *ahk3-3* mutant, upon exogenous DHZ treatments, showed no sensitivity, with no reduction in root length or meristem size even after 24 h of treatment (Figure 5 and Supplemental Figures S1–S3).

**Figure 6:**
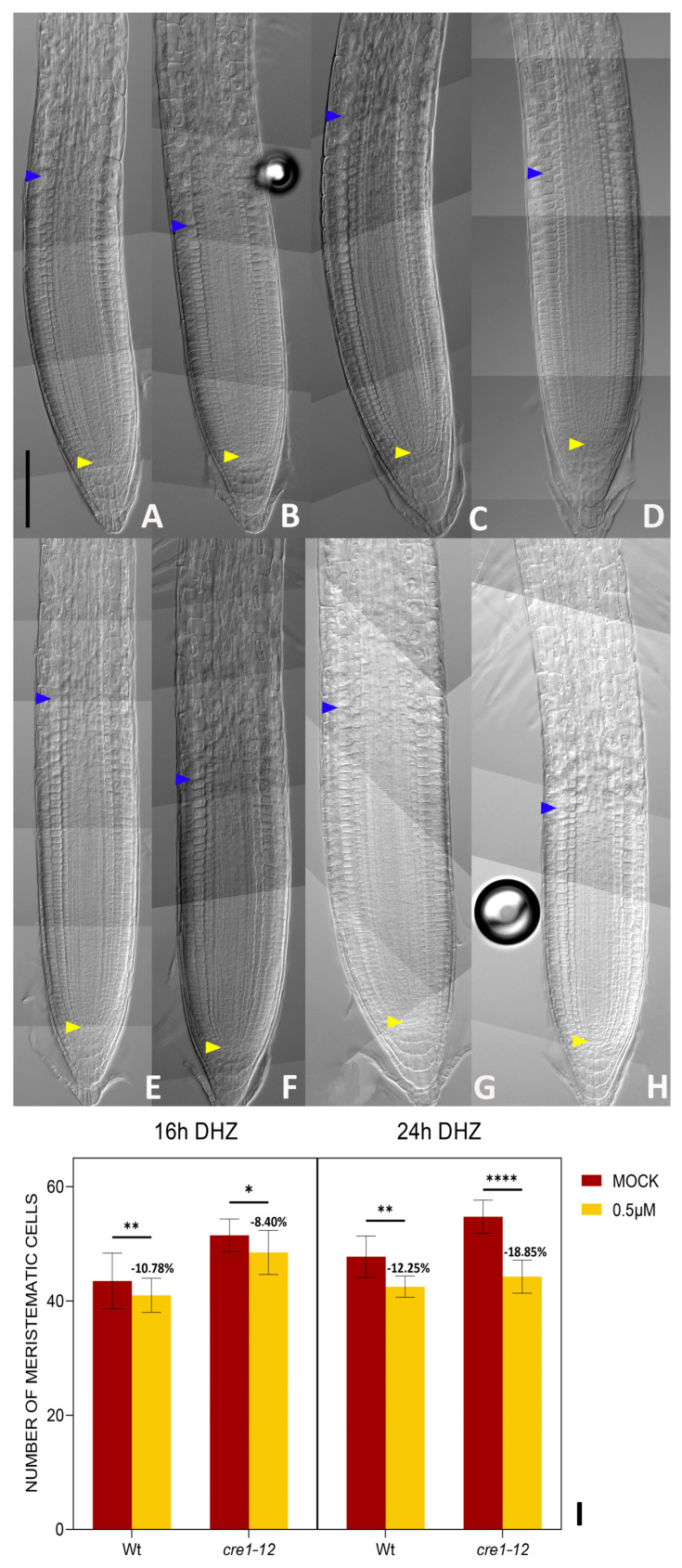
CRE1 does not mediate DHZ activity in the root meristem. (**A**–**D**) Representative DIC optical microscope images of 6 dpg Wt and *cre1-12* plants. Scalebar = 100 μm. The roots were mock-treated with EtOH (**A**–**C**) or treated for 16 h with 0.5 μM DHZ (**B**–**D**). Yellow arrows indicate the QC, blue arrows indicate the last cortical cell of the meristem. Scalebar = 100 μm. (**E**–**H**) Representative DIC optical microscope images of 6 dpg Wt and *cre1-12* plants. Scalebar = 100 μm. The roots were mock-treated with EtOH (**E**–**G**) or treated for 24 h with 0.5 μM DHZ (**F**–**H**). Yellow arrows indicate the QC, blue arrows indicate the last cortical cell of the meristem. Scalebar = 100 μm. (**I**) Analysis of meristematic cortical cell number of Wt and *cre1-12* plants treated for 16 h with 0.5 μM DHZ. Mock treatment: EtOH. Error bars indicate standard deviation (SD). (*) indicates a significance with a *p*-value < 0.05, (**) indicates a significance with a *p*-value < 0.01, (****) indicates a significance with a *p*-value < 0.001, two-way ANOVA with Tukey’s post hoc test, N= 3, n = 10.

In contrast, upon *tZ* treatments, the *ahk3-3* mutant displayed a slight reduction in root meristem size after 24 h, suggesting that the mutant is slightly sensitive to *tZ* (Figure 5 and Supplemental Figures S1–S3). Among cytokinin receptors, only *CRE1* and *AHK3* are strongly expressed at the TZ, while *AHK2* is primarily expressed in the stem cell niche (Supplemental Figure S4). Consistent with this expression pattern, *ahk2-5* mutant does not exhibit an enlarged number of meristematic cells relative to Wt plants (Supplemental Figure S5). *AHK3* is expressed in all tissues of the TZ, while *CRE1* is restricted to the vasculature of the meristem and of the TZ ([31], Supplemental Figure S4). To investigate if DHZ promotes cell differentiation, also acting via CRE1, we treated a *CRE1* loss-of-function mutant (*cre1-12*) with DHZ. The *cre1-12* mutant exhibited similar sensitivity to DHZ as Wt plants, phenocopying their response (Figure 6 and Supplemental Figures S1–S3). These data suggest that AHK3, but not CRE1, is part of the signal transduction mechanism triggered by DHZ controlling root meristem size.

### 2.3. DHZ Regulates TZ Positioning via ARR1, 11 and 12

**Figure 7:**
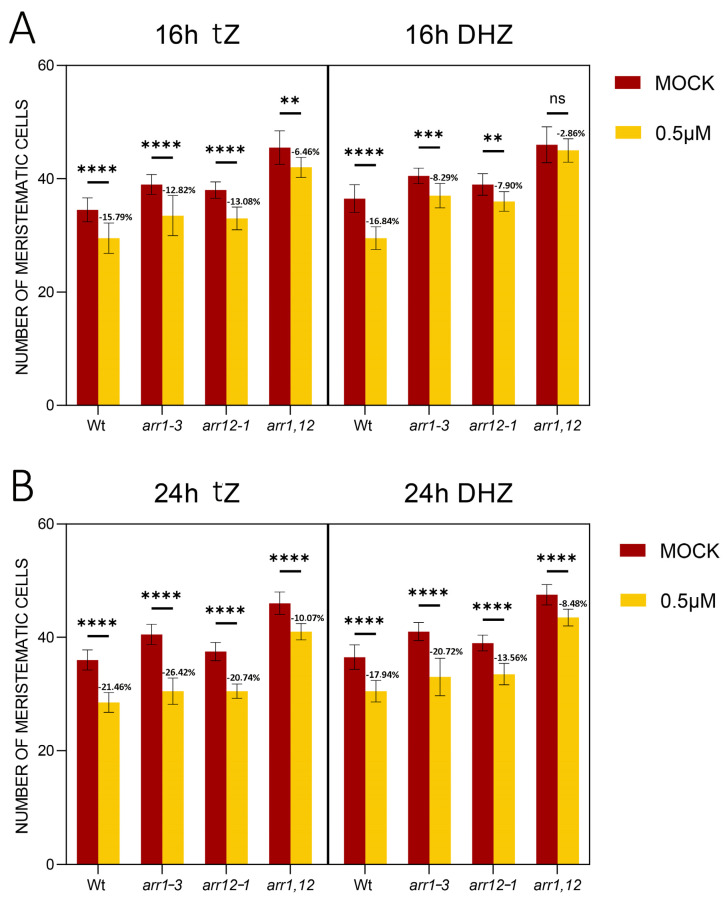
Mutations in *ARR1* and *12* are not sufficient to confer resistance to *tZ* and DHZ in the root meristem. (**A**) Analysis of the meristematic cortical cell number of Wt, *arr1-3*, *arr12-1*, and *arr1,12* plants treated for 16 h with 0.5 μM *tZ* or 0.5 μM DHZ. Mock treatments: DMSO for *tZ*, EtOH for DHZ. Error bars indicate standard deviation (SD). (ns) indicates a *p*-value > 0.05, (**) indicates a significance with a *p*-value < 0.01, (***) indicates a significance with a *p*-value < 0.005, (****) indicates a significance with a *p*-value < 0.001, two-way ANOVA with Tukey’s post-hoc test, N = 3, n = 15. Representative root images are reported in the Supplemental Figure S6. (**B**) Analysis of the meristematic cortical cell number of Wt, *arr1-3*, *arr12-1*, and *arr1,12* plants treated for 24 h with 0.5 μM *tZ* or 0.5 μM DHZ. Mock treatments: DMSO for *tZ*, EtOH for DHZ. Error bars indicate standard deviation (SD). (****) indicates a significance with a *p*-value < 0.001, two-way ANOVA with Tukey’s post-hoc test, N = 3, n = 15. Representative root images are reported in the Supplemental Figure S7.

**Figure 8:**
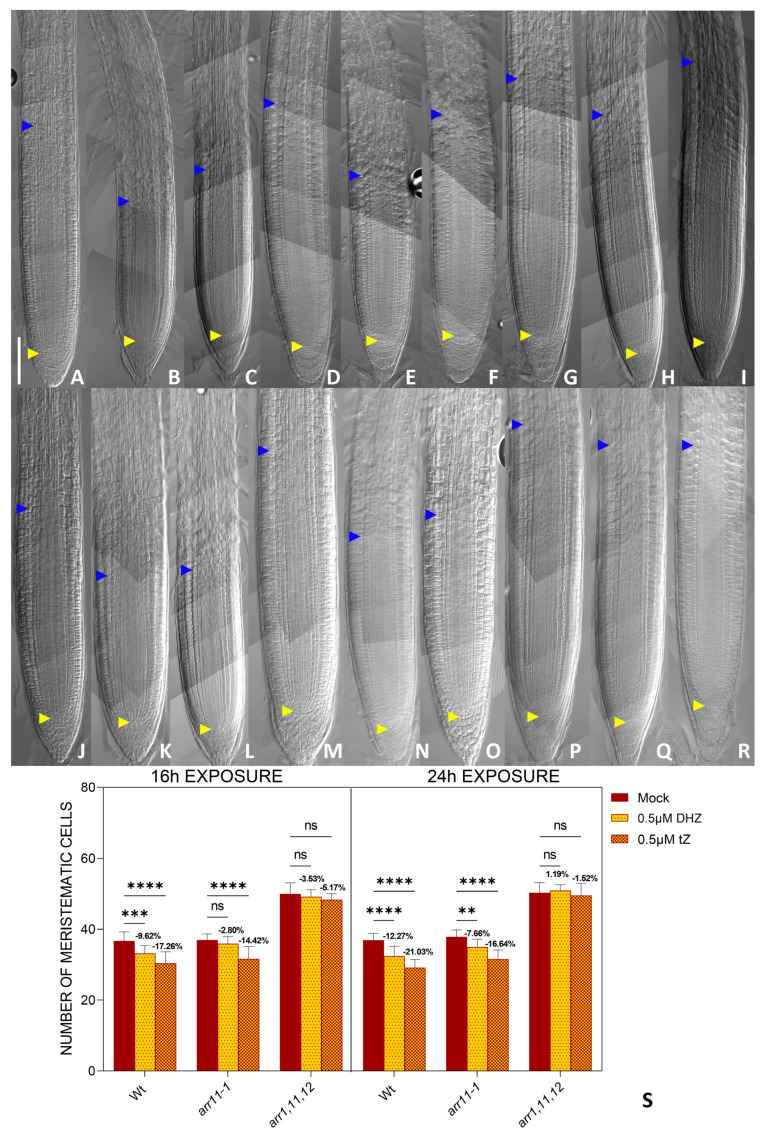
ARR11 cooperates with ARR1 and 12 to regulate root meristem size. (**A**–**I**) Representative DIC optical microscope images of 6 dpg Wt (**A**–**C**), *arr11-1* (**D**–**F**) *arr1*, *11*, and *12* (**G**–**I**) plants. The roots were mock-treated with water (**A**,**D**,**G**) or treated for 16 h with either 0.5 μM *tZ* (**D**,**E**,**H**) or 0.5 μM DHZ (**C**,**F**,**I**). Yellow arrows indicate the QC, blue arrows indicate the last cortical cell of the meristem. Scalebar = 100 μm. (**J**–**R**) Representative DIC optical microscope images of 6 dpg Wt (**J**–**L**), *arr11-1* (**M**–**O**) *arr1*, *11*, and *12* (**P**–**R**) plants. The roots were mock-treated with water (**J**,**M**,**P**) or treated for 24 h with either 0.5 μM *tZ* (**K**,**N**,**Q**) or 0.5 μM DHZ (**L**,**N**,**R**). Yellow arrows indicate the QC, blue arrows indicate the last cortical cell of the meristem. Scalebar = 100 μm. (**S**) Analysis of meristematic cortical cell number of Wt, *arr11-1*, and *arr1,11,12* plants treated for 16 h with 0.5μM DHZ or 0.5μM *tZ*. Mock treatment: water. Error bars indicate standard deviation (SD). (ns) indicates a *p*-value > 0.05, (**) indicates a significance with a *p*-value < 0.01, (***) indicates a significance with a *p*-value < 0.005, (****) indicates a significance with a *p*-value < 0.001, one-way ANOVA with Tukey’s post hoc test, N = 3, n = 10.

Upon perception of the *tZ*, AHK3 promotes cell differentiation, activating the transcription factors Type B ARR 1 and 12 transcription factors [56]. To understand if this was the case also upon activation of AHK3 mediated by DHZ, we treated the single *arr1-3* and *arr12-1* and double *arr1*, *arr12* loss of function mutants with DHZ. As previously reported these mutants show enlarged root meristem size [56]. When exposed for 16 hrs to DHZ *arr1-3* and *arr12-1* single mutants and *arr1,12* double mutants display no reduction in root meristem size, suggesting that these genes are activated also upon AHK3-dependent DHZ perception. However, prolonged exposure (24 h) of the mutants to both DHZ and *tZ* eventually results in a slight reduction in root length and meristem (Figure 7 and Supplemental Figures S1, S2, and S7). Considering that *ahk3-3* mutants are insensitive to DHZ, these data suggest that additional type-B ARRs might be activated by AHK3 after DHZ and *tZ* perception to induce cell differentiation. Recent single-cell sequencing data reported that the type-B ARR11 is expressed in the TZ (Rootcellatlas.org). We thus wondered whether this gene is also involved in the AHK3-dependent downstream signaling pathway. *arr11-1* single mutants show no root length or root meristem phenotypes; however, triple loss of function mutant *arr1,arr12,arr11* show increased root length and meristem size in comparison to *arr1,12* double mutants (Figure 8). Treatments of *arr1*, *arr12*, and *arr11* plants with *tZ* or DHZ for 24 h showed that this mutant combination is insensitive to both tZ and DHZ (Figure 8 and Supplemental Figures S1, S2 and S6–S8). These results suggested that ARR11 cooperates with ARR1 and 12 in controlling root meristem activity upon AHK3-dependent *tZ* and DHZ perception.

### 2.4. Root Meristem Size Regulation Operated by DHZ Is Potentially Conserved Among Plants

**Figure 9:**
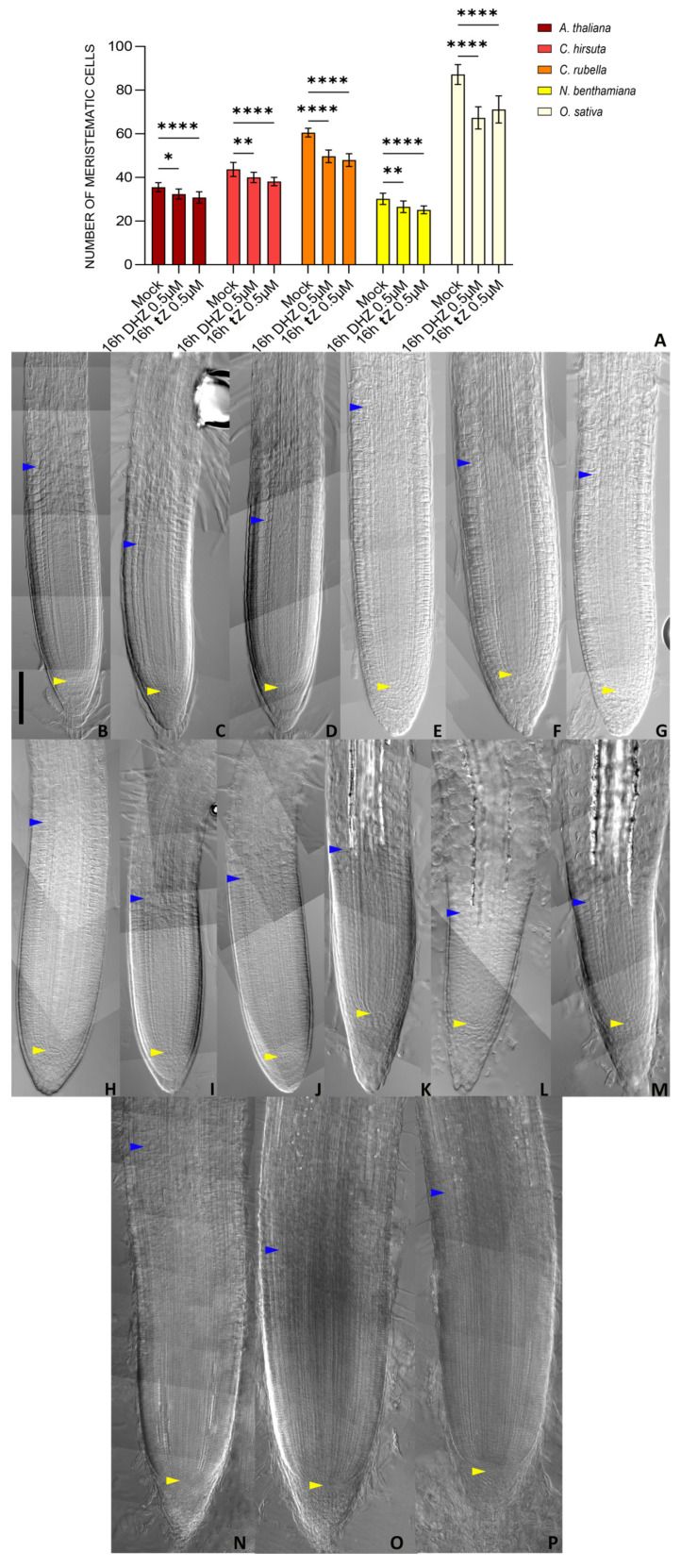
DHZ and *tZ* regulate root meristem size in a conserved manner. (**A**) Analysis of meristematic cortical cell number of *Arabidopsis*, *Cardamine*, *Capsella*, *Nicotiana*, and *Oryza* Wt plants treated for 16 h with 0.5 μM DHZ or 0.5 μM *tZ*. Mock treatment: water. Error bars indicate standard deviation (SD). (*) indicates a significance with a *p*-value < 0.05, (**) indicates a significance with a *p*-value < 0.01, (****) indicates a significance with a *p*-value < 0.001, one-way ANOVA with Tukey’s post-hoc test, N = 3, n = 10. (**B**–**P**) Representative DIC optical microscope images of 6 dpg *Arabidopsis* (**B**–**D**), *Cardamine* (**E**–**G**), *Capsella* (**H**–**J**), *Nicotiana* (**K**–**M**), and *Oryza* (**N**–**P**) plants. The roots were mock-treated with water (**B**,**E**,**H**,**K**,**N**) or treated for 16 h with either 0.5 μM *tZ* (**C**,**F**,**I**,**L**,**O**) or 0.5 μM DHZ (**D**,**G**,**J**,**M**,**P**). Yellow arrows indicate the QC, blue arrows indicate the last cortical cell of the meristem. Scalebar = 100 μm.

Most of the experiments regarding cytokinin regulation of root development have been performed in the plant model system *Arabidopsis*. We wondered whether the pro-differentiation activity of DHZ and tZ is conserved in both short- and long-term evolutionary scales. To this end, we treated with *tZ* and DHZ *Capsella rubella* and *Cardamine hirsuta* seedlings, two close relatives of *Arabidopsis* belonging to the Brassicaceae family, and analysed their root meristem sizes. Because *Cardamine* and *Capsella* have two cortical layers [57], we counted the meristem cell number of the outer cortical layer starting from the QC up to the first elongated cell. Similarly to *Arabidopsis*, we noticed that exposure for 16 h to 0.5 uM *tZ* or DHZ was sufficient to decrease the root meristem size in both species (Figure 9). This data suggests a conserved DHZ and *tZ* activity in the control of the root meristem size in short timescale evolution.

To understand if DHZ and *tZ* can control root meristem size on a larger evolutionary scale, we analyzed the effects of treatments of *tZ* and DHZ on the root meristem of the phylogenetically distant plant *Nicotiana benthamiana*, a Solenaceae model system. Similar to *Capsella* and *Cardamine*, *Nicotiana* root anatomy is characterized by multiple cell layers of the cortex tissue [58]. We applied the same approach used for meristem size analysis in the other species. After 16 h of treatment with 0.5 µM DHZ and *tZ*, *Nicotiana* exhibited a reduced meristem size compared to untreated plants (Figure 9). Finally, to explore whether *tZ* and DHZ could regulate root meristem size in monocots, we analyzed the root meristem development in response to these two compounds in the monocot model plant *Oryza sativa* (rice). As rice shows a complex root architecture, we focused our attention on the radicle, as it is comparable to dicot primary roots [58,59,60,61,62,63,64,65]. For rice, we analyzed the number of cells of the outermost cortical layer next to the epidermis, starting from the QC up to the first elongated cell. We noticed that, similarly to what we reported for the tested dicots, also in rice *tZ* and DHZ applications, can reduce the radicle meristem size. Overall, these data support the hypothesis that *tZ* and DHZ may regulate root development of flowering plants in a conserved fashion over an evolutionary timescale.

## 3. Discussion

The pro-differentiative role of cytokinins in the *Arabidopsis* root apical meristem has been widely investigated [26,42]. Indeed, the AHK3/ARR1/ARR12 pathway has been untangled extensively, and the role of CRE1 in the vasculature development is well characterized [38,66,67]. Nonetheless, the majority of the results obtained via pharmacological treatments were acquired by using *tZ*, considered the most active cytokinin [68]. Although some evidence hints at the fact that these compounds are perceived by the plant and trigger physiological responses, not much has been done to unravel their contribution to developmental processes and to disentangle the downstream signalling pathways [11]. Aside from tZ, DHZ is another common and naturally occurring isoprenoid cytokinin [68]. Biochemical and heterologous expression experiments have shown that the AHK3 receptor binds DHZ, whereas tZ is perceived by both AHK3 and CRE1 [29]. Here we propose that DHZ is a cytokinin that actively promotes root cell differentiation in an AHK3-dependent pathway. Consistent with the described role, DHZ is sufficient to promote the transcription of the cell differentiation effectors *KRP2*, *SHY2*, and *GH3.17*. In particular, we showed in vivo that DHZ is perceived similarly to tZ in the root Transition Zone but differently from the latter; DHZ is bound only by AHK3 and not by the CRE1 receptor.

We show that upon perception, DHZ, as well as *tZ*, can activate three different types B ARRs: ARR1, 12, and ARR11, which we identified and related to the promotion of root cell differentiation.

Hence, based on our results, we posit that the AHK3 and CRE1 cytokinin receptors regulate root meristem homeostasis in an additive manner. CRE1 is largely involved in developmental programmes patterning the vascular tissue, where AHK3’s role seems marginal [67]. Consistent with this, *ahk3* mutants show phenotypic defects in the vascular tissue only when in combination with *cre1* [67]. Since in *Arabidopsis tZ* is converted into DHZ by an unidentified zeatin reductase, we propose that *tZ* serves as the primary cytokinin regulating developmental processes, while DHZ may play a more specific role, particularly in the context of cell differentiation. The isolation of the enzymatic pathway prompts the formation of DHZ from tZ, and the characterization of the spatio-temporal regulation of such a pathway from a developmental perspective will help clarify this crucial point. Several stresses are able to promote degradation of cytokinin, triggering the expression of CYTOKININ OXIDASE (CKX) genes [69,70]. Interestingly, it has been reported that CKX enzymes are not able to catabolize DHZ [68,71]. Hence, DHZ activity might play a crucial role in promoting root cell differentiation in response to stresses, maintaining defined root patterning and regulating growth. Future studies will help to clarify this aspect.

Notably, the role of both *tZ* and DHZ in root meristem homeostasis regulation might be conserved among flowering plants. Indeed, DHZ and *tZ* treatments can decrease the root meristem size also in close relatives of *Arabidopsis*, such as *Cardamine hirsuta* and *Capsella rubella*, as well as in distant species, such as *Nicotiana benthamiana* or *Oriza sativa*. Although we do not know if the activity of *tZ* and DHZ across species is exerted by orthologues of the AHK3/ARR1/12/11 module, the analysis of mutants for these orthologous genes and the generation of higher-order mutants will contribute to clarifying this point. Additionally, the high affinity of AHK3 to bind DHZ might be a prerogative of *Arabidopsis*. In support of this possibility, it has been shown in potato, maize, tobacco, rapeseed, and rice that most of the HK receptors show affinity for DHZ [72,73,74].

Altogether, our suggestion is that different types of cytokinins could play specific roles in development. Similar conclusions have already been suggested by studies on cis-Zeatin, a tRNA-derived cytokinin [75]. Different from DHZ and *tZ*, this type of cytokinin promotes cell division in the root meristem via yet unidentified pathways. Double mutants of two ISOPENTENYL TRANSFERASE genes that regulate only cis-Zeatin biosynthesis, *ipt2* and *ipt9* show a shorter root meristem than the Wt [76], a phenotype that is opposite to other mutants in cytokinin signaling and biosynthesis genes.

Further studies are necessary to understand whether and how different zeatin types control development. Nonetheless, our findings suggest that different types of cytokinins may preferentially bind to specific receptors to establish developmental programs. Based on our data, this study lays the foundation for investigating the diverse developmental roles of the different molecules within the same class of hormones.

## 4. Conclusions

**Figure 10:**
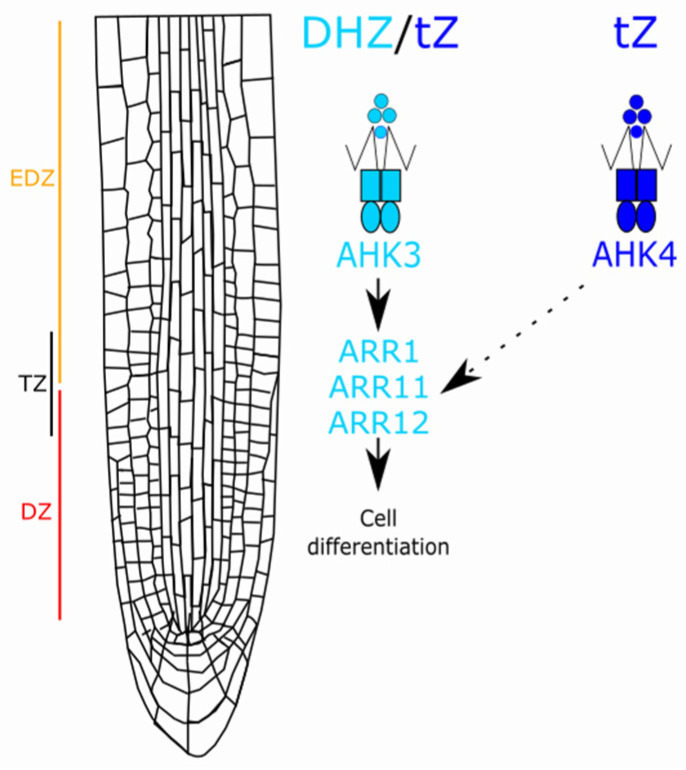
DHZ and tZ bind to AHK3 to promote the activity of the type-B ARRs ARR1-11-12, and in turn cell differentiation in the root. Differently, AHK4 binds only tZ and might activate cell differentiation via the same ARRs.

Our findings shed light on the specific role of DHZ in root cell differentiation. Different from tZ, which can control several aspects of root development, DHZ specifically acts as a pro-differentiation input, triggering the AHK3-ARR1, 11, and 12 dependent pathways (Figure 10). Hence, we propose a model in which cell differentiation activity is finely regulated by the selective binding of different zeatin types to specific receptors. Furthermore, the conserved pro-differentiation effect of DHZ and tZ across multiple plant species suggests a plausible evolutionarily conserved mechanism governing root meristem development. The identification of the enzymatic pathway responsible for DHZ biosynthesis and the characterization of key differentiation effectors across species will be a crucial step in deciphering the evolutionary conservation of the cytokinin-dependent pro-differentiation mechanisms. Overall, our study provides a foundation for understanding how different types of cytokinins contribute to plant developmental plasticity, opening new roads for the study of hormone-regulated differentiation processes.

## 5. Materials and Methods

### 5.1. Plant Material and Growth Conditions

*Arabidopsis thaliana* accession line *Columbia-0 (Col-0)* was used as wild-type for experimentations, as well as wild type *Cardamine hirsuta (Oxford)*, *Capsella rubella*, *Nicotiana benthamiana*, *Oryza sativa (Nipponbare)* plants. All *Arabidopsis* mutants and transgenic lines are in the Col-0 background, as detailed. Mutants *ahk3-3* and plants containing *pAHK3::AHK3:GUS* or *pCRE1::CRE1:GUS* were provided by Tatsuo Kakimoto (Osaka University, Osaka, Japan). *arr1-3*, *arr11-1*, and *arr12-1* plants were provided by Mike Mason (University of Queensland, Brisbane, Australia) [77]. *gh3.17–1* and *cre1-12* mutants were obtained from the Nottingham *Arabidopsis* Stock Centre collection (SALK_050597 and SALK_048970C, respectively). Homozygous mutants from the Salk T-DNA were identified by PCR as described (http://signal.salk.edu/). *pSHY2::SHY2:GUS* and *pKRP2::KRP2:GUS* expressing plants were provided by [56] and [44], respectively. Plants expressing *pGH3.17::3xYFP* and *pGH3.17::GH3.17:GFP* were provided by Riccardo Di Mambro (Università di Pisa, [43]). Plants expressing *pRCH2::3xYFP* and *pTCS::nGFP* were provided by [44] and [78], respectively. Higher-order mutants were obtained by crossing the above single mutant lines and screening the progeny via genotyping/PCR to identify homozygous plants. Seeds were sterilized as described by [79], and seedlings were grown on one-half strength Murashige and Skoog (MS) medium containing 0.8% agar at 22 °C in long-day conditions (16-h-light/8-h-dark cycle) as previously described by [80] (the only exception is *N. benthamiana*, grown on complete MS medium).

### 5.2. Arabidopsis Locus IDs from This Article

ARR1 (AT3G16857), ARR11 (AT1G67710), ARR12 (AT2G25180), AHK3 (AT1G27320), CRE1 (AT2G01830), GH3.17 (AT1G28130), IAA3/SHY2 (AT1G04240), and KRP2 (AT3G50630).

### 5.3. Meristem-Size Analysis

The number of meristematic cortex cells was measured as described by [81]. Root meristem size of 6 days post germination plants was computed as the number of cortex cells, counting the first cell after the quiescent center as the first meristematic cell, and the cell preceding the first elongated cortex cell as the last one, as described [80]. Cells were counted using a Differential Interference Contrast (DIC) microscopy equipped with Nomarski technology (Zeiss Axio Imager A. Plants were mounted in a chloral hydrate solution (8:3:1 mixture of chloral hydrate:water:glycerol).

### 5.4. Analysis of Expression Patterns

β-glucoronidase activity of transgenic plants was visualized after a 30 min vacuum treatment and a minimum of 2 h staining. GFP and 3xYFP signals were imaged using a confocal laser scanning microscope (Zeiss LSM780; Zeiss, Oberkochen, Germany). The cell wall was stained with 10 μM propidium iodide. The quantification of the fluorescence of the reporter lines was performed using the open-source software ImageJ V1.53 (https://imagej.net/ij/, accessed on 19 April 2025). Upon image calibration, the area of interest was selected with the polygon tool, then the raw integrated density (RawIntDen) and the area of the selection were measured, and the value of the fluorescence was represented by the ratio between the RawIntDen and the ROI area. The obtained values were then analyzed statistically as described in the figure legends. The analyses were performed with a sufficiently large number of samples to ensure statistical significance.

### 5.5. Hormonal Treatments

Hormones were prepared as follows: *tZ* (Duchefa) dissolved in DMSO and DHZ (Duchefa) in EtOH. Diluted in water to uniform mock treatments.

5 dpg seedlings were transferred from solid one-half MS medium to solid one-half MS medium supplemented with hormones or mock (DMSO for *tZ* and EtOH for DHZ). In cases where the solvent for hormones was not water, an appropriate quantity of solvent was added to the medium for mock treatments.

### 5.6. Image Processing and Assembly

The acquired microscopy images were processed and analyzed with FIJI/ImageJ (https://imagej.net/ij/). For root length computation, the plates were photographed (6 dpg), and the root length was measured as the distance from the root shoot junction to the root tip with the segmented line selection tool at different timeframes. Figure assembly was performed with Adobe Photoshop 2020. Image post-processing was minimal and restricted to corrections in brightness and contrast or assembly of multiple snaps. All corrections were uniformly applied to the entire image so that all final figures properly reflect the original data

### 5.7. Quantification and Statistical Analysis

Statistical analysis was performed using GraphPad V9.0 (www.graphpad.com). A sufficiently large number of samples to ensure statistical significance was used to perform every analysis, as reported in the corresponding figures. In every figure legend, details regarding statistical tests used and error bars are provided for the represented experiment. Representative pictures of the experiments were presented in every figure and in the Supplemental Data. N indicates the number of biological replicates, while n indicates the number of samples for each replicate. For our experiments, all the outcomes of the replicated experiments were consistent with each other, and one replicate is presented in every figure.

## Data Availability

The original contributions presented in this study are included in the article/Supplementary Materials. Further inquiries can be directed to the corresponding author.

## Supplementary Materials

The following supporting information can be downloaded at: https://www.mdpi.com/article/10.3390/plants14101501/s1, Figure S1: *tZ* and DHZ affect root growth; Figure S2: Mock treatment does not alter *ahk3*, *arr1*, *arr12* root meristem size; Figure S3: AHK3 mediates DHZ activity. Figure S4: CRE1 controls *TZ* position. Figure S5:. AHK2 is not involved in positioning the *TZ*.; Figure S6: ARR1 and 12 are not sufficient to mediate DHZ activity. Figure S7: ARR1 and 12 are not sufficient to mediate DHZ activity in the root meristem. Figure S8: DHZ requires ARR1, 11, and 12 to position the *TZ*. Figure S9: Mock treatments do not alter Wt, *arr11-1*, and *arr1*, *11*, *12* root meristem size.
