# Supplementary material for: Identification of a Specific Role of Dihydrozeatin in the Regulation of the Cell Differentiation Activity in Arabidopsis Roots
Source: Plants (Basel). 2025 May 16;14(10):1501. doi: 10.3390/plants14101501 (PMC12114884; doi:10.3390/plants14101501)
Supplement: Supplementary file 1 [file plants-14-01501-s001.zip › plants-3538843-supplementary.pdf]

SUPPLEMENTAL FIGURES/DATA:

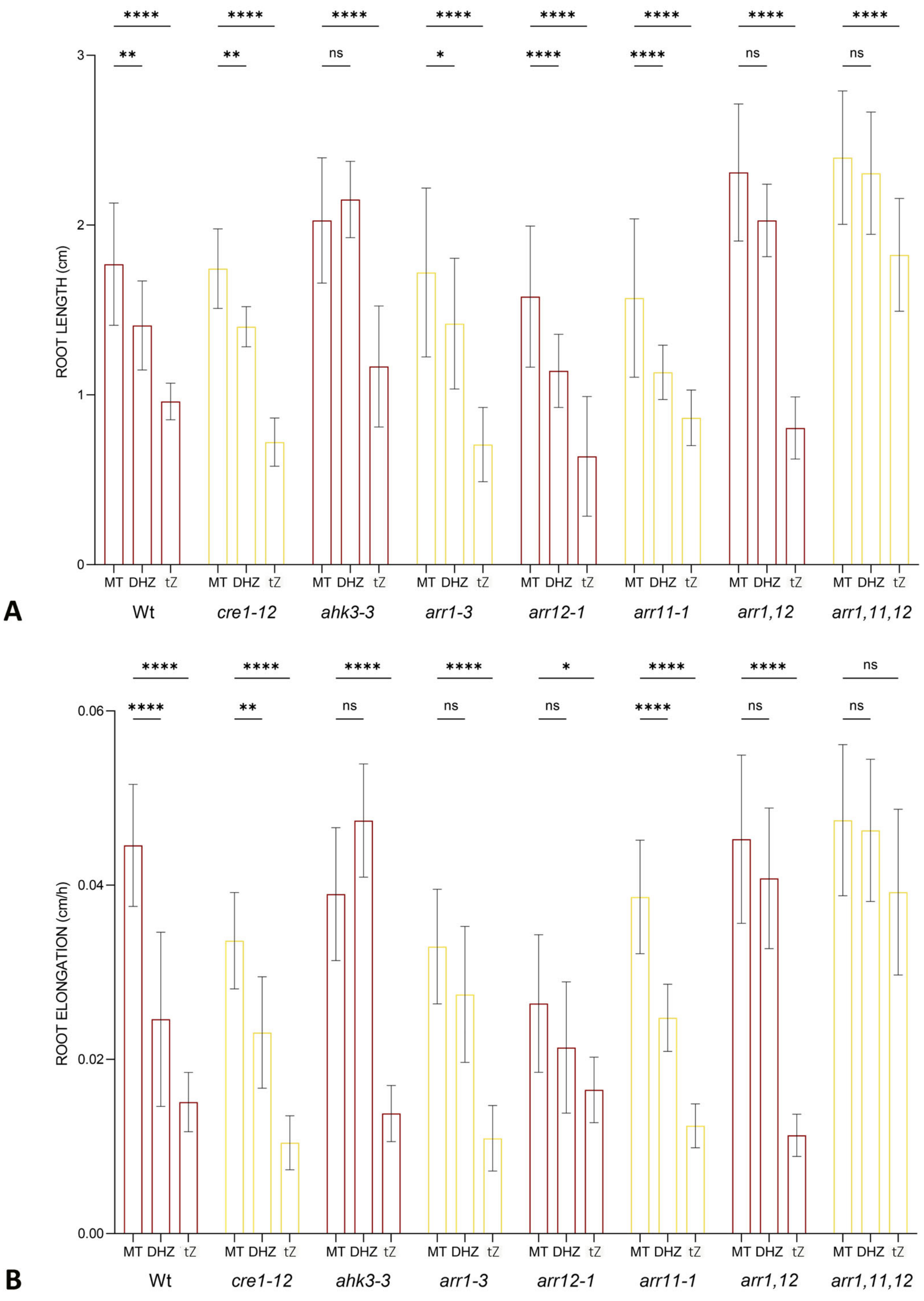

Supplemental Figure S**1:** *tZ* and DHZ affect root growth.

A) Root length of 5dpg seedlings exposed to CKs since germination. B) Growth velocity of 5dpg seedlings exposed to CKs since germination. MT stands for mock treated. Error bars indicate standard deviation (SD). (ns) indicates lack of significance, (*) indicates a significance with a p-value < 0.05, (**) indicates a significance with a p-value < 0.01, (***) indicates a significance with a p-value < 0.005, (****) indicates a significance with a p-value < 0.001, Student's t-test, N=3, n = 15.

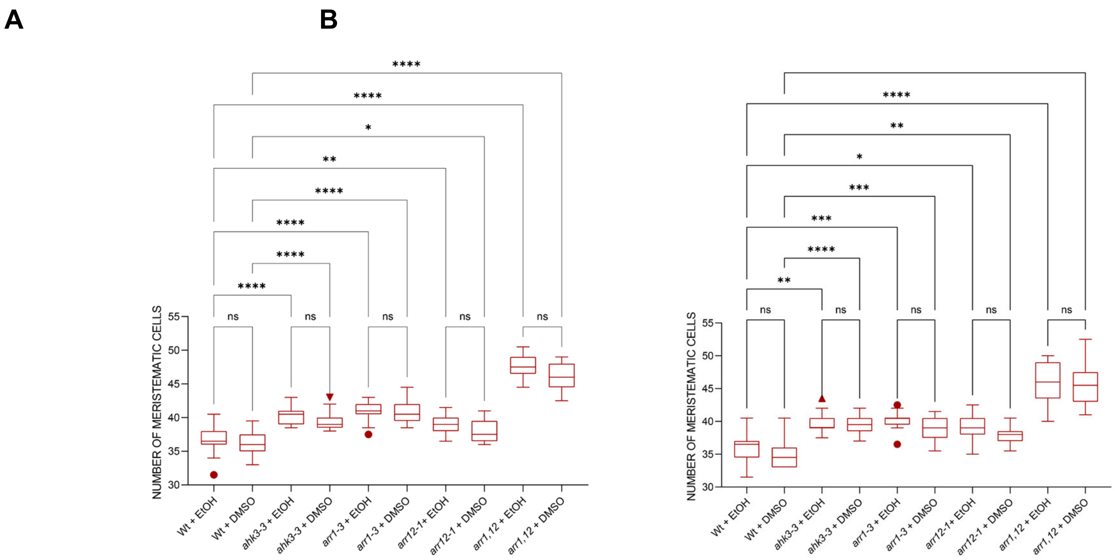

Supplemental Figure S2: Mock treatment does not alter *ahk3*, *arr1*, *arr12* root meristem size. (A) 16h exposition to mock. Error bars indicate standard deviation (SD). (*) indicates a significance with a p-value < 0.05, one-way Anova with Tukey's post-hoc test, N=3, n = 15. (B) Analysis of meristematic cortical cell number of Wt, *ahk3-3*, *arr1-3*, *arr12-1* and *arr1,12* mock treated plants. 24h exposition to mock. Error bars indicate standard deviation (SD). (ns) indicates lack of significance, (*) indicates a significance with a p-value < 0.05, (**) indicates a significance with a p-value < 0.01, (***) indicates a significance with a p-value < 0.005, (****) indicates a significance with a p-value < 0.001,, one-way Anova with Tukey's post-hoc test, N=3, n = 15.

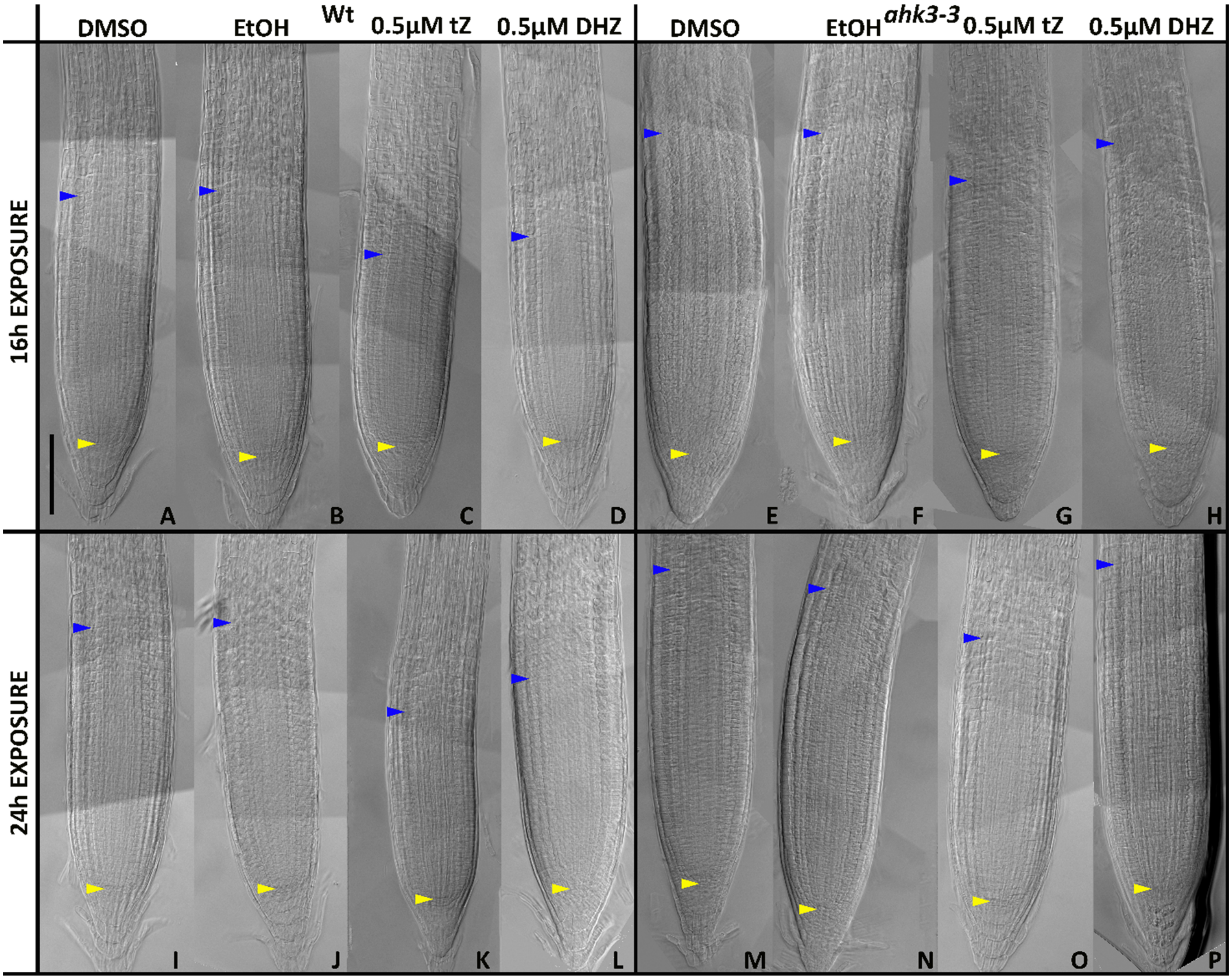

Supplemental Figure S3: AHK3 mediates DHZ activity. Representative DIC optical microscope images of 6dpg Wt (A-D, I-l) and *ahk3-3* (E-H, M-P) plants, related to analysis in Figure 6. The roots were exposed to treatments for 16 (A-H) or 24 hours (I-P). Plants were mock treated with DMSO (A-E-I-M) or ethanol (B,F,J,N), or treated with either 0.5µM *tZ* (C,G,K,O) or 0.5µM DHZ (D,H,L,P). Yellow arrows indicate the QC, blue arrows indicate the last cortical cell of the meristem. Scalebar = 100µm.

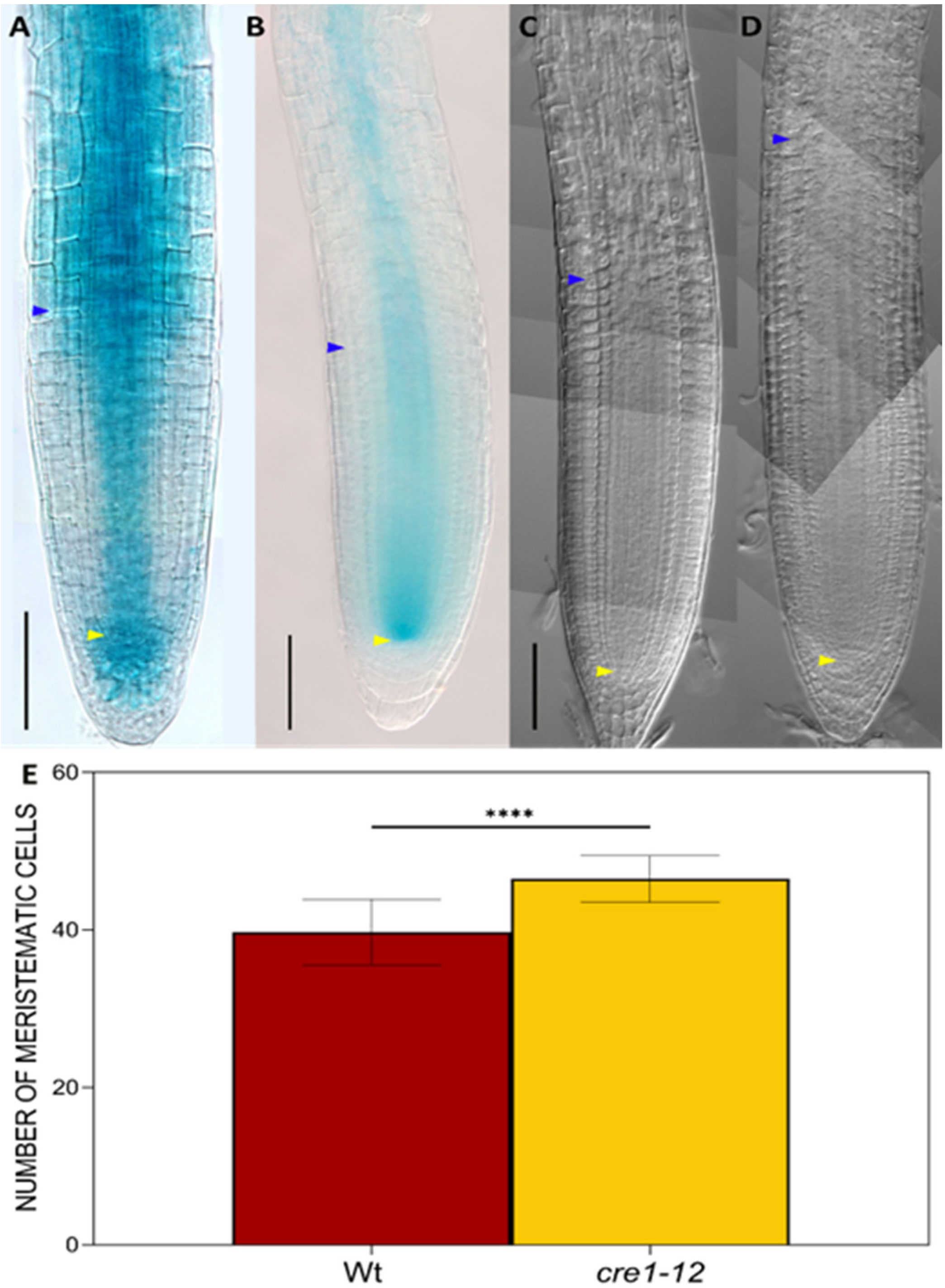

Supplemental Figure S4**:** CRE1 controls *TZ* position.

A-B) Histochemical GUS assay of *Arabidopsis* root. Representative DIC optical microscope images of 6dpg transgenic plants carrying a *AHK3::GUS* (A) or *AHK4::GUS* (B) fusion. Scalebars = 100µm. N=3, n=15.

C-D) Representative DIC optical microscope images of 6dpg Wt and *cre1-12* plants. Scalebar = 100µm.

E) Analysis of meristematic cortical cell number of Wt and *cre1-12*. Error bars indicate standard deviation (SD). (****) indicates a significance with a p-value < 0.001, Student's t-test, N=3, n = 15.

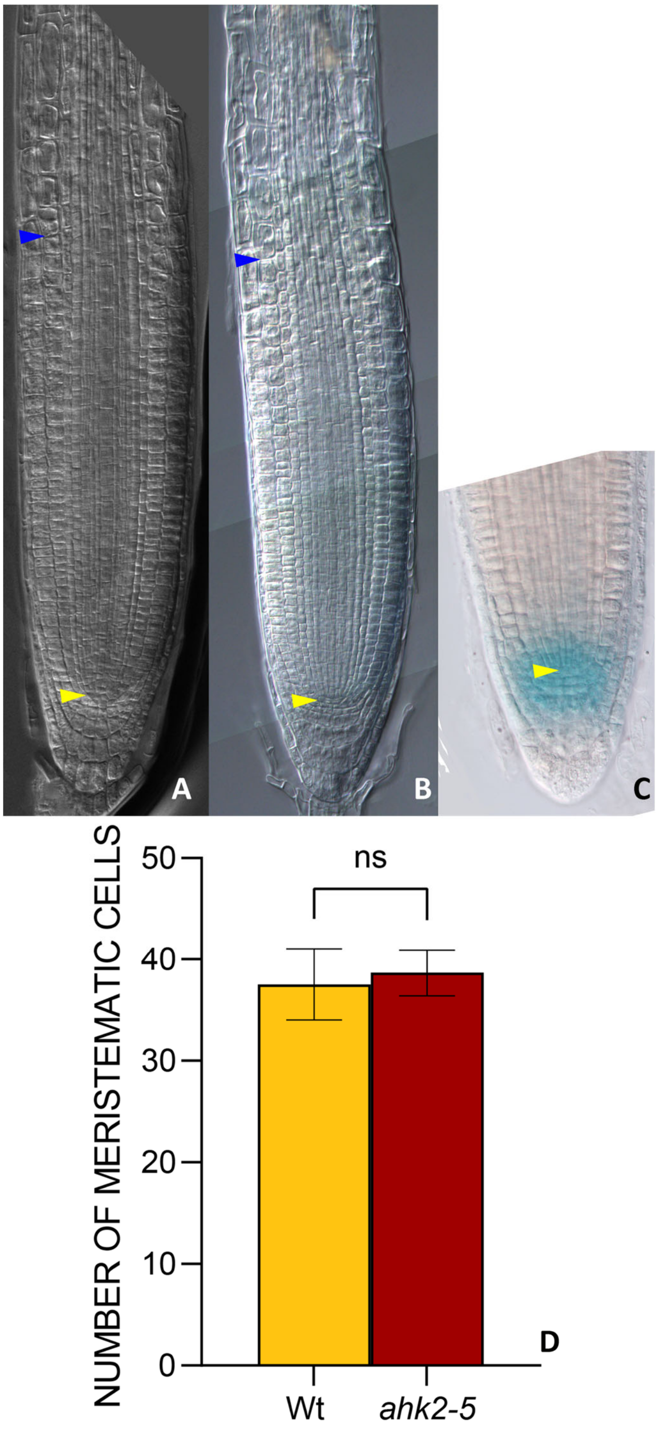

Supplemental Figure S5**:** AHK2 is not involved in positioning the *TZ*. A-B) Representative DIC optical microscope images of 6dpg Wt and *ahk2-5* plants. Scalebar = 100µm. . N=3, n=15. C) Histochemical GUS assay of *Arabidopsis* root. Representative DIC optical microscope image of 6dpg transgenic plants carrying a *AHK2::GUS* fusion. Scalebar = 100µm. N=3, n=15. E) Analysis of meristematic cortical cell number of Wt and *ahk2-5*. Error bars indicate standard deviation.(SD), (ns) indicates lack of significance.

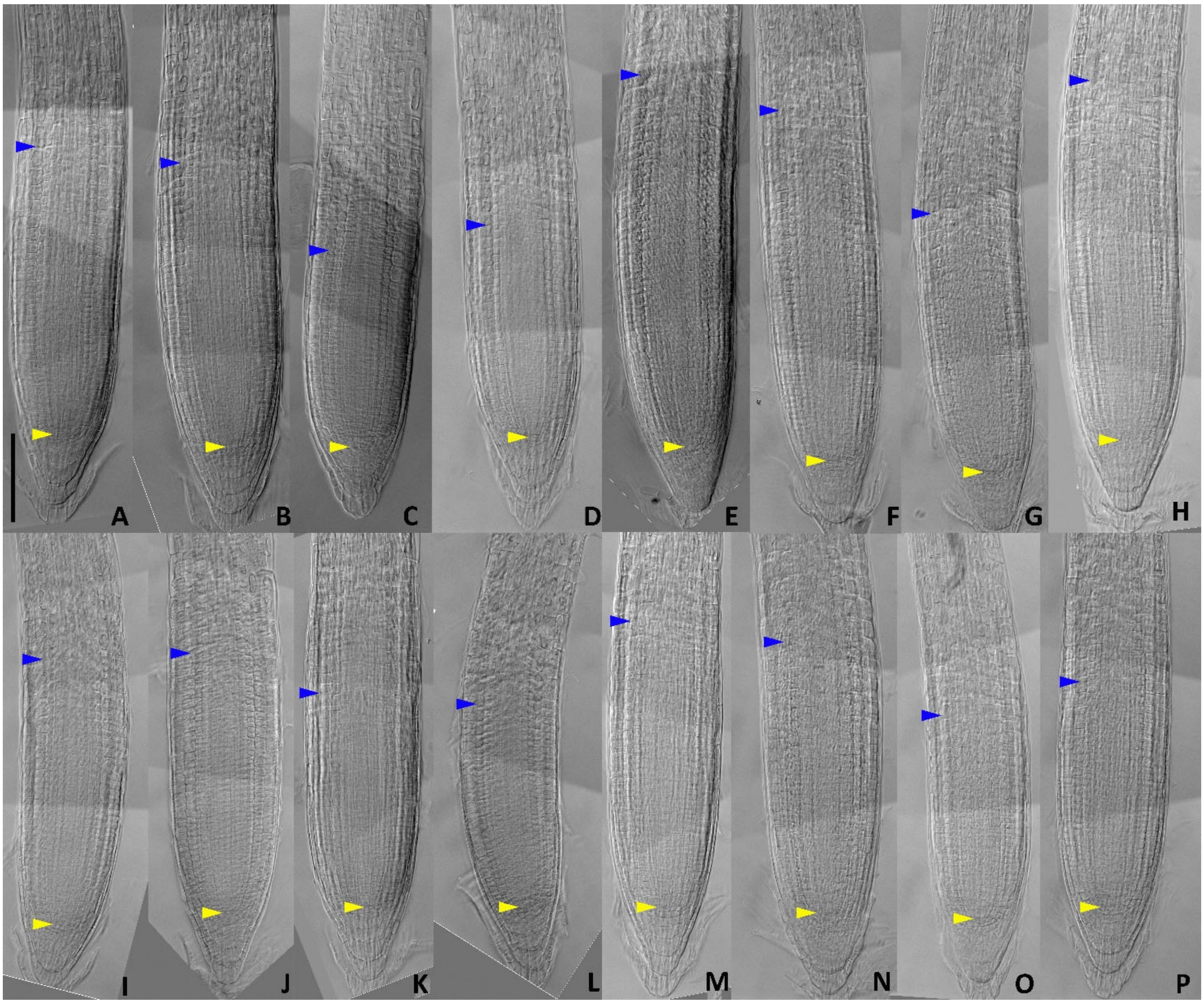

Supplemental Figure S6: ARR1 and 12 are not sufficient to mediate DHZ activity. Representative DIC optical microscope images of 6dpg Wt (A-D), *arr1,12* (E-H), *arr12-1* (I-L) and *arr1-3* (M-P) plants, related to analysis in Figure 7A. The roots were exposed to treatments for 16h. Plants were mock treated with DMSO (A-E-I-M) or ethanol (B,F,J,N), or treated with either 0.5µM *tZ* (C,G,K,O) or 0.5µM DHZ (D,H,L,P). Yellow arrows indicate the QC, blue arrows indicate the last cortical cell of the meristem. Scalebar = 100µm.

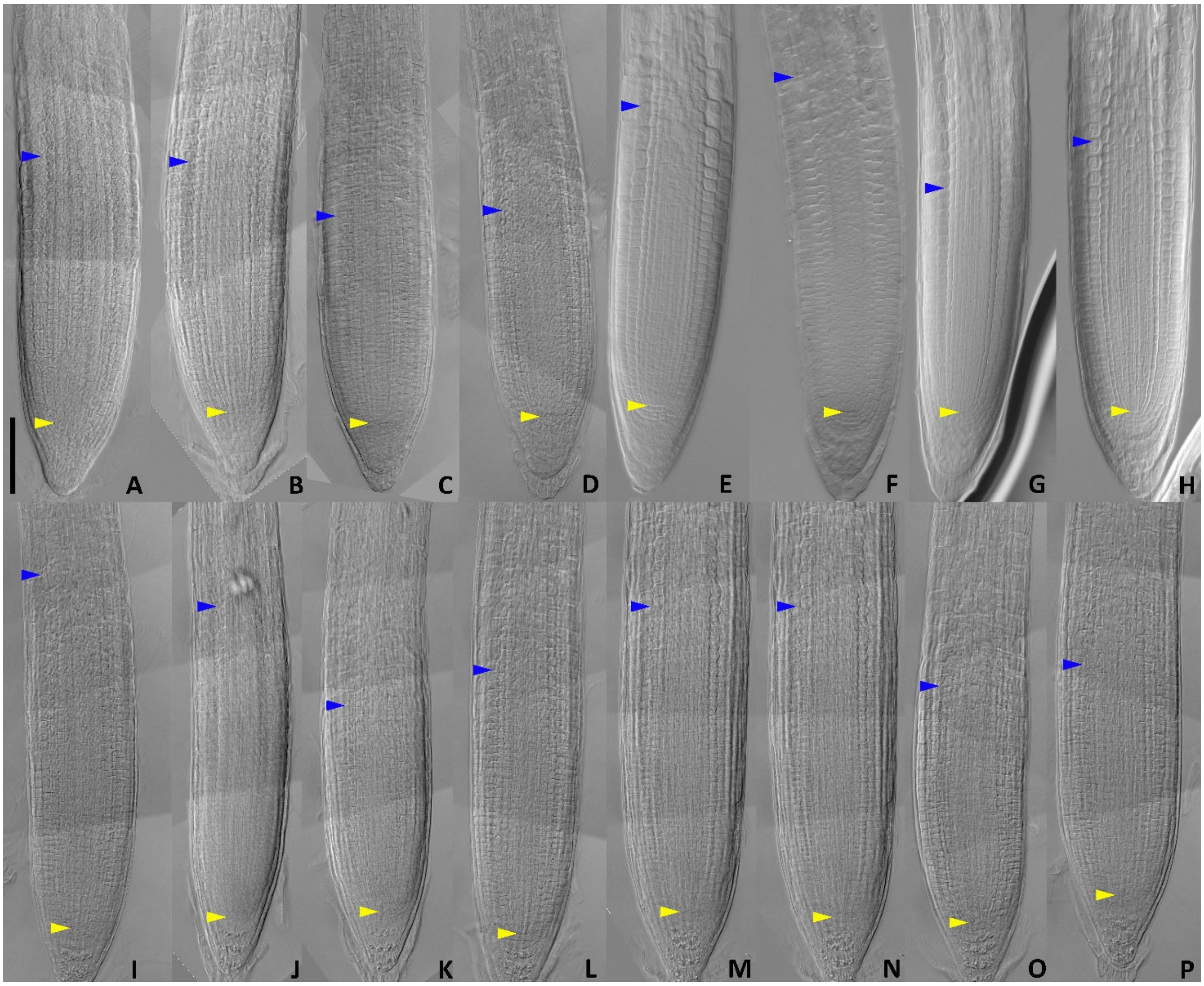

Supplemental Figure S7: ARR1 and 12 are not sufficient to mediate DHZ activity in the root meristem. Representative DIC optical microscope images of 6dpg Wt (A-D), *arr1,12* (E-H), *arr12-1* (I-L) and *arr1-3* (M-P) plants, related to analysis in Figure 7B. The roots were exposed to treatments for 24h. Plants were mock treated with DMSO (A-E-I-M) or ethanol (B,F,J,N), or treated with either 0.5µM *tZ* (C,G,K,O) or 0.5µM DHZ (D,H,L,P). Yellow arrows indicate the QC, blue arrows indicate the last cortical cell of the meristem. Scalebar = 100µm.

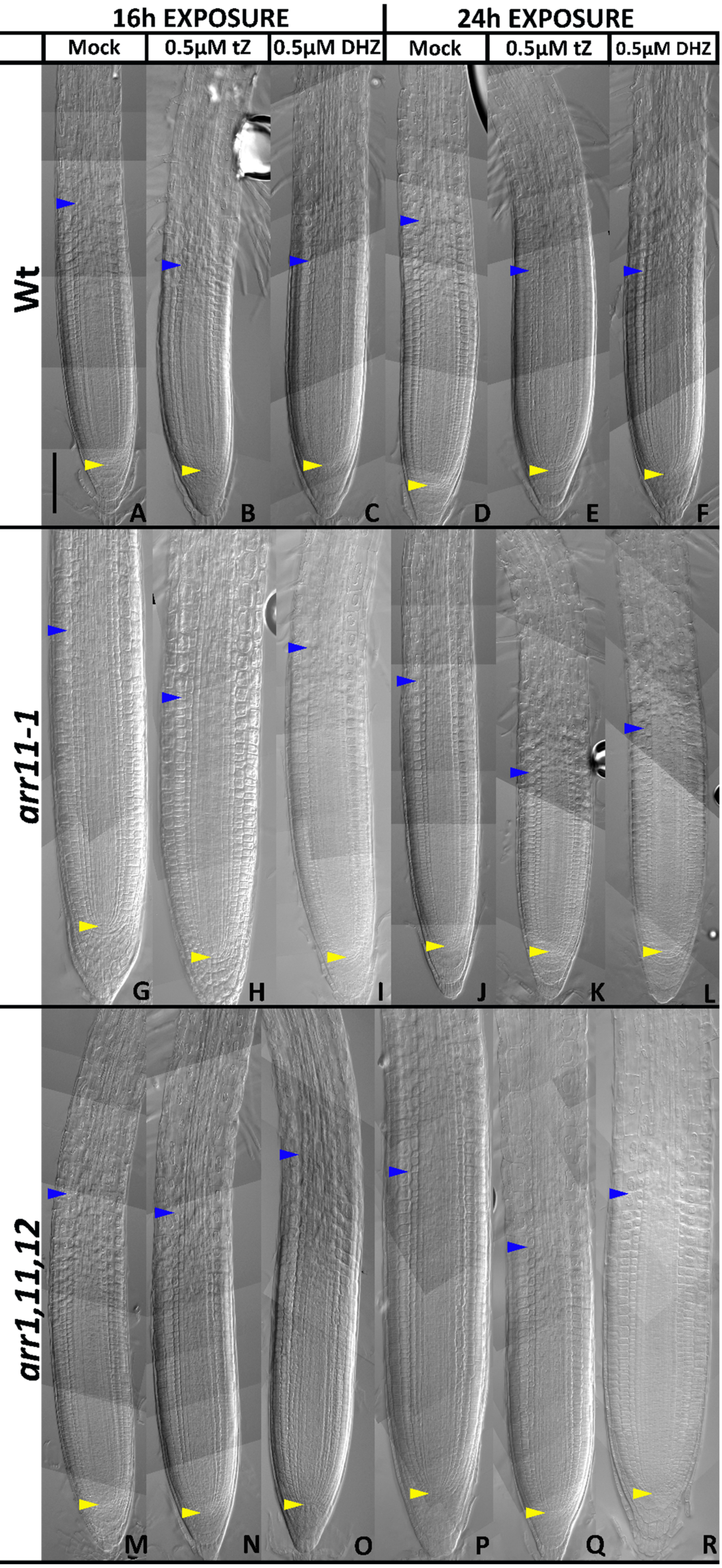

Supplemental Figure S8: DHZ requires ARR1,11 and 12 to position the *TZ*. Representative DIC optical microscope images of 6dpg Wt (A-F), *arr11-1* (G-L) and *arr1,11,12* (M-R) plants, related to analysis in Figure 8. The roots were exposed to treatments for 16h or 24h. Yellow arrows indicate the QC, blue arrows indicate the last cortical cell of the meristem. Scalebar = 100μm.

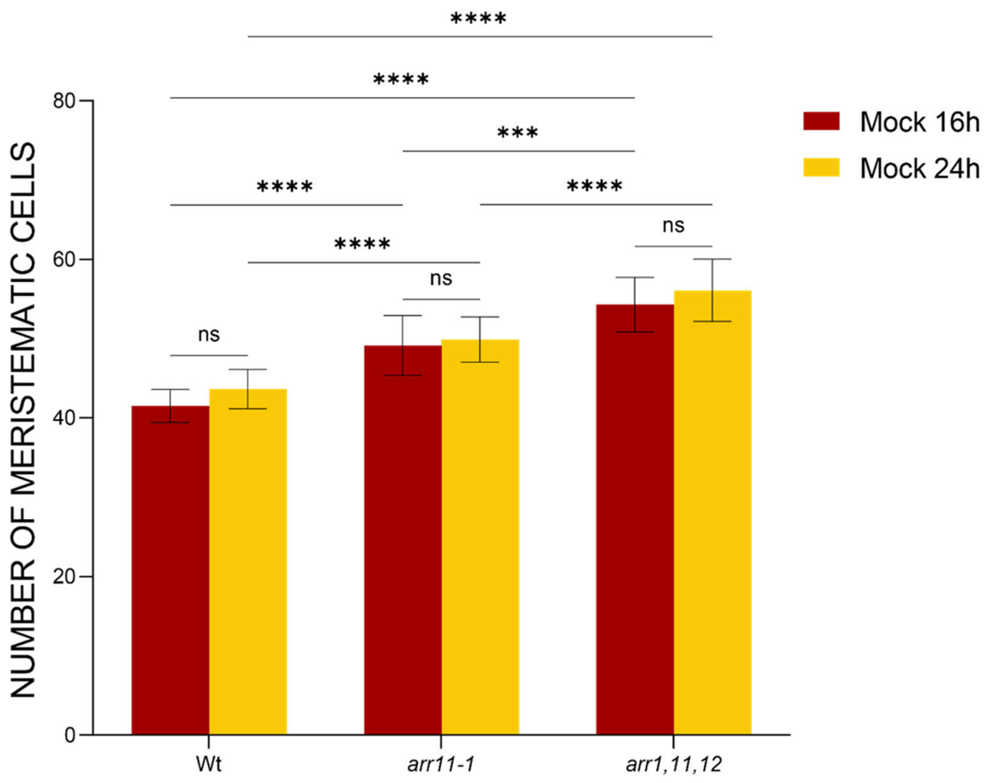

Supplemental Figure S9: Mock treatments do no alter Wt, *arr11-1* and *arr1,11,12* root meristem size (related to figure 8). 16h or 24h exposition to mock. Error bars indicate standard deviation (SD). (ns) indicates lack of significance, (***) indicates a significance with a p-value < 0.005, (****) indicates a significance with p-value < 0.001, one-way Anova with Tukey's post-hoc test, N=3, n = 15.
